# Huntingtin exon 1 deletion does not alter the subcellular distribution of huntingtin and gene transcription in mice

**DOI:** 10.3389/fncel.2022.1021592

**Published:** 2022-11-10

**Authors:** Xianxian Zhao, Yize Sun, Zhifu Wang, Laiqiang Chen, Shihua Li, Xiao-Jiang Li

**Affiliations:** Guangdong Key Laboratory of Non-human Primate Research, Guangdong-Hongkong-Macau Institute of CNS Regeneration, Jinan University, Guangzhou, China

**Keywords:** polyglutamine, Huntington, autophagy, gene expression, subcellular distribution

## Abstract

Huntington disease (HD) is caused by the expansion of CAG triplet repeats in exon 1 of the *huntingtin (HTT)* gene, which also encodes the first 17 amino acids (N-17) that can modulate the toxicity of the expanded polyQ repeat. N-17 are conserved in a wide range of species and are found to influence the subcellular distribution of mutant Htt. Moreover, N-17 is subject to many posttranslational modifications that may regulate the function, stability, and distribution of HTT. However, the function of Htt exon 1 and its influence on the normal Htt remains to be fully investigated. By investigating a knock-in mouse model that lacks *Htt* exon1, we found that deletion of *Htt* exon1 does not affect the survival of mice and differentiation of cultured mouse neurons. Furthermore, the lack of *Htt* exon 1 does not alter the subcellular distribution of Htt, autophagy protein expression, and global gene transcription in the mouse brain. These results suggest that removing the entire exon 1 of Htt could be a therapeutic approach to eliminate expanded polyQ toxicity.

## Introduction

Huntington’s disease (HD) is a neurodegenerative disorder with an autosomal dominant trait and is caused by unusual expansion of CAG DNA triplet repeats in exon 1 of the IT15 gene, which is translated to an expanded polyglutamine (polyQ) repeat in huntingtin (HTT), a 350-kDa protein whose function remains to be fully investigated (MacDonald et al., 1993; Saudou and Humbert, 2016). The expanded polyQ repeat in mutant HTT contains more than 36 glutamines and leads to a progressive loss of motor coordination and function, chorea, and eventually death within 10 to 15 years after the onset of the disease in midlife (Ross and Tabrizi, 2011; Bates et al., 2015). Despite widespread distribution of mutant HTT throughout the body and brain, mutant HTT causes selective neuronal loss in the brain with the preferential death of GABAergic medium-sized spiny neurons in the neostriatum and large neurons in layer VI of the cerebral cortex (Ross and Tabrizi, 2011; Bates et al., 2015).

Like other eight polyglutamine diseases, including SCA 1, 2, 3, 6, 7, 17. Dentatorubral-pallidoluysian atrophy (DRPLA), and spinobulbar muscular atrophy (SBMA), HD currently has no cure. Because mutant HTT can affect a variety of cellular functions, eliminating mutant HTT would be the most effective way to treat HD (Caron et al., 2018; Tabrizi et al., 2020). To this end, gene editing has been used to remove the expanded CAG repeats that is localized in exon 1 of HTT or to selectively delete the mutant allele (Liu et al., 2016; Yang et al., 2017; Dabrowska et al., 2018). However, it would be challenging to just remove the polyQ domain without disrupting N-terminal HTT. On the other hand, removing the exon 1, which consists of a N17 domain and polyQ followed by PRR (Proline-rich region) domain, would be less difficult, but it remains to be investigated whether the exon 1 of HTT is an indispensable part for the essential function of HTT.

Previous studies have shown that HTT exon 1 plays a significant role in membrane anchoring, protein interaction, and protein complex formation (Li and Li, 2004; Michalek et al., 2013; Tao et al., 2019; Vieweg et al., 2021). N17 amino acids in exon 1 are well conserved across species and are thought to be important for HTT’s function as well as the toxicity of mutant HTT. In support of this, N17 amino acids can markedly modify the toxicity of mutant HTT and its nuclear localization (Cornett et al., 2005; Gu et al., 2009; Arndt et al., 2019; Chiki et al., 2021; Vieweg et al., 2021).

However, growing evidence indicates that mice lacking polyQ domain and most part of exon 1 of HTT do not show obviously abnormal growth and behaviors (Zheng et al., 2010; André et al., 2017; Yang et al., 2020; Braatz et al., 2021). Given that HTT is a multifaced protein and is essential for the early development of mice (Saudou and Humbert, 2016), it is important to investigate whether HTT’s normal function is dependent on its N-terminal exon 1 region, and addressing this issue is important for treating HD.

In this study, we used a knock-in mouse model with deletion of the mouse *Htt* exon 1 to examine the subcellular distribution of Htt, neuronal development, autophagy protein expression, and gene transcription. Our results show no significant differences between wild type mice and mutant mice without *Htt* exon 1, suggesting that exon 1 in *Htt* gene can be safely removed to eliminate neurotoxicity of the expanded polyQ in treating HD.

## Materials and Methods

### Mouse maintenance and breeding

Mice (C57BL/6) were bred and maintained in the animal facility at Jinan University under specific pathogen-free conditions in accordance with institutional guidelines of the Animal Care and Use Committee at Jinan University. All mice were maintained on a 12:12 h light/dark cycle (lights off at 9 p.m.). The temperature was maintained at 22 ± 1°C with relative humidity (30%–70%). The studies followed the protocol approved by the Animal Care and Use Committee at Jinan University. To stress mice, starvation was induced by removing food for exactly 24 h, with sufficient water supply. The D177 mice, with a deletion of 177 bp in exon 1 of *Htt* gene, was generated in our previous study (Yang et al., 2020). The gRNA sequences used to target the mouse *Htt* exon 1 were (PAM site in lowercase): T1: cccTGGAAAAGCTGATGAAGGC; T3: ccaGGTCCGGCAGAGGAACCGC, and the primers used to genotype KI mice were: S1, ACTGCTAAGTGGCGCCGCGTAG (mouse genomic HTT 98–118) and A1, AGGAGGTAACCCTAGAGATCT CTGC (mouse genomic HTT 700–724; Yang et al., 2017).

### Cell culture and immunofluorescence

Striatal neurons were obtained from the brain tissues of P1 (postnatal day 1) mouse. The brain tissues were treated with HBSS (Ca^2+^ free and homemade) containing 0.125 mg/ml Trypsin (Gibco^TM^ #25200056) and then passed through 1 ml tip for 20 times, followed by centrifugation of the cells at 1,000× *g* for 5 min. Cells were resuspended in the medium (Neurobasal A BRL#10888-022 with 0.4 mM L-glutamine BRL#25030-081, 1% fetal bovine serum, 2% B27 Gibco^TM^ #A3582801, and 1% penicillin-streptomycin mixture Gibco^TM^ #15140122) and then set in the tissue culture plates pre-coated with 0.1 mg/ml poly-D-lysine. After 14 days, the primary cultured neurons were fixed with 4% paraformaldehyde in PBS (phosphate buffered saline, 137 mM NaCl, 2.7 mM KCl, 10 mM NaH_2_PO_4_ and 2 mM KH_2_PO_4_) for 10 min at room temperature. The fixed cells were washed with PBS for three times (5 min each time), and then blocked in 3% BSA/2% donkey serum/0.2% Triton X-100 in PBS for 1 h. After washes with PBS three times, the cells were incubated in blocking buffer with 0.1% Triton X-100 and primary antibody at 4°C overnight. The primary antibody was then removed, and the cells were washed with PBS, followed by adding secondary antibody (in 3% BSA/PBS) with DAPI (4’,6-Diamidino-2-phenylindole dihydrochloride, Sigma #D9542) onto the cells for 30 min at 4°C before examination.

### Western blotting analysis

For Western blotting, mouse brain tissue was dissected and lysed in ice-cold RIPA lysis buffer (50 mM Tris, pH 8.0, 150 mM NaCl, 1 mM EDTA pH 8.0, 0.1% SDS, 0.5% Sodium Deoxycholate and 1% triton X-100) containing halt protease inhibitor cocktail (ThermoScientific #78429), 2 mM Na_3_VO_4_, 10 mM NaF, and PMSF. The lysates were incubated on ice for 30 min and then sonicated for 10 s. For western blotting analysis, equal amounts of protein from the whole lysates were resolved by 4%–20% or 4%–12% gels (BeyoGel^TM^ SDS-PAGE Precast Gel, #P0056 and #P0057) or 4% or 6% homemade tris-glycine SDS-PAGE gels for detecting full-length Htt. The proteins in SDS gel were transferred to a nitrocellulose membrane (0.2 μm, Millipore #ISEQ00010) that was then incubated with appropriate primary antibodies using the dilution as indicated in Table 1).

**Table 1:** Antibodies used in the study.

**Antibody**	**Host**	**Dilution**	**Catalog No.**	**RRID**	**Supplier**	**Application**
Huntingtin	Mouse	1:1,000	MAB2166	RRID:AB_11213141	Millipore	WB
Huntingtin	Mouse	1:5,000	EPR5526	RRID:AB_10863082	Abcam	WB
Huntingtin	Rabbit	1:1,000	D7F7	RRID:AB_10827977	Cell Signaling	IF
NeuN	Mouse	1:500	MAB377	RRID:AB_2298772	Millipore	ICC
TUJ1	Mouse	1:500	MAB1637	RRID:AB_2210524	Millipore	ICC
MEK1/2	Mouse	1:1,000	4,694	RRID:AB_10695868	Cell Signal Tech.	WB
Histone H3	Rabbit	1:1,000	GTX122148	RRID:AB_10633308	GeneTex	WB
RPS6	Rabbit	1:1,000	GTX113542	RRID:AB_2037923	GeneTex	WB
Calnexin	Rabbit	1:1,000	GTX109669	RRID:AB_1949824	GeneTex	WB
MBP	Rat	1:500	MAB386	RRID:AB_94975	Millipore	WB
PSD95	Rabbit	1:1,000	3,450	RRID:AB_2292883	Cell Signal Tech.	WB
VDAC1	Rabbit	1:10,000	ab15895	RRID:AB_2214787	Abcam	WB
Beclin 1	Rabbit	1:1,000	3,495	RRID:AB_1903911	Cell Signal Tech.	WB
P62	Mouse	1:1,000	ab56416	RRID:AB_945626	Abcam	WB
ATG16L	Rabbit	1:1,000	8,089	RRID:AB_10950320	Cell Signal Tech.	WB
pATG16L	Rabbit	1:1,000	84,300	RRID:AB_2915984	Cell Signal Tech.	WB
LAMP1	Rabbit	1:1,000	Ab108597	RRID:AB_2915985	Abcam	WB
LC3B	Rabbit	1:2,000	Ab192890	RRID:AAB_2827794	Abcam	WB
Vinculin	Rabbit	1:10,000	Ab129002	RRID:AB_11144129	Abcam	WB
GAPDH	Mouse	1:10,000	Ab8245	RRID:AB_2107448	Abcam	WB

WB, Western blotting; ICC, immunocytochemistry; IF, immunofluorescence.

### Subcellular fractionation and Western blotting

The whole fresh brains of D177 and wild-type mouse were minced with a pair of scissors to 2–4 nm pieces, resuspend in 6 volumes of solution A in a homogenizer (Glass, loose type) and homogenized in 0.25 M sucrose, 2 mM Tris-HCl pH 7.4, 1 mM MgCl_2_, 100 mM NaCl, 1 mM K_2_HPO_4_ pH 7.0, 1 mM EDTA, 1× protease inhibitor, phosphates inhibitors (2 mM sodium orthovanadate , Na_3_VO_s_, and 10 mM Sodium Fluoride NaF) with 25 strokes. After passing a 27G needle 10 times, the homogenate was centrifuged at 700× *g* for 10 min at 4°C to yield the pellet, P1, and the supernatant S1. The S1 was then centrifuged at 15,000× *g* for 30 min at 4°C to yield S2 and P2, and the P2 was put on the top of 0.32/0.8/1.0/1.2 M sucrose gradients with 2 mM Tris-HCl pH 7.4, 0.15 M NaCl, 1 mM MgCl_2,_ and 1 mM EDTA, followed by centrifugation (Beckman SW32 rotor, #355631 tube) at 25,000 rpm for 2 h at 4°C. After centrifugation, LP1, LP2, LP3, and LP4 were isolated from layers between 0.32–0.8 M, 0.8–1.0 M, 1.0–1.2 M, and the pellet, respectively. S2 was centrifuged at 100,000× *g* for 1 h to yield P3 and S3. S3 was centrifuged at 185,000× *g* for 2 h to provide P4 and S4 (Type Ti 100 rotor).

To compare the distribution of Htt in each fraction, we used Western blotting to analyze the subcellular distribution of Htt by examining its level in the total homogenates and all fractions, and each fraction was adjusted according to the relative volume in the “total”.

### Immunofluorescence

The mice were anesthetized with 1% isoflurane and perfused with 0.9% NaCl, followed by 4% paraformaldehyde (PFA). The brains were removed and fixed in 4% PFA overnight at 4°C and dehydrated in 30% sucrose at 4°C for 48 h. The brains were then cut to 20 μm sections with a cryostat at −20°C. We used HTT antibody D7F7 for HTT immunofluorescence staining, and the slices were incubated in sodium citrate buffer (10 mM sodium citrate, 0.05% Tween 20, pH 6.0) at 95°C for 10 min for antigen retrieval. The slices were then blocked with 2% donkey serum, 3% BSA and 0.3% Triton-X 100 in PBS for 2 h and incubated with primary antibodies in the same buffer at 4°C overnight. After washing with 1× PBS, the sections were incubated in fluorescent secondary antibodies. Fluorescent images were acquired with a Zeiss microscope (Carl Zeiss imaging, Axiovert 200 MOT).

### RNA-sequencing

Animals were euthanized using isoflurane (>5%) to dissect the cortex, striatum, and cerebellum. All brain tissues were shipped in dry ice to Biomarker Technologies, in Beijing, for performing the RNA-sequencing analysis. Total RNA (1 μg) was used to construct sequencing libraries enriched by magnet beads with Oligo (dT) and randomly fragmented using fragmentation buffer. The RNA fragments were amplified using random hexamers, end repaired and adenylated, and then sequenced using Illumina platform.

The RNA-seq data was first mapped and quantified using salmon software (ver 1.8.0) on the high-performance computing platform at Jinan University, and the quant data was next counted with edgeR to explore differentially expressed genes (DEGs; Robinson et al., 2010). The top 1,000 DEGs ranked by *q*-values were displayed on the heatmap using the ComplexHeatmap R package (ver 2.13.1; Gu et al., 2016). The significant up-regulate and down-regulate genes (*p* < 0.01, |Fold Change| > 1) in different brain regions between D117 and WT mice were shown on the volcano plot through the EnhanceVolcano R package (ver 1.14.0; Blighe et al., 2019). To estimate different pathway activation scores in samples, the neuronal-associated pathways sets were chosen and Poplawski et al. was referred to and calculated through Gene Set Variation Analysis (GSVA) with GSVA R package (ver 1.44.2; Hänzelmann et al., 2013; Poplawski et al., 2020). The HTT interacting proteins were chosen according to literature (Li and Li, 2004) and the FPKM values of genes were recalculated to Z-score and plotted on the heatmap. All above analysis were performed on the R (ver 4.6.0) and R studio (ver 2022.07.01, Build 554).

### Statistics

The mice carrying D177 genotype were calculated using Hardy Weinberg equilibrium. For Western blotting analysis, at least three independent experiments were performed and the relative levels of proteins on the blots were obtained using the ratios of density of interesting bands to the loading control band (vinculin). Statistical significance of Western blotting was assessed using Student’s *t*-test or multiple Student’s *t*-test for comparing two groups to determine statistical significance. Data are mean ± SEM. Calculations were performed with GraphPad Prism software (7.04).

## Results

### Deletion of *Htt* exon 1 did not affect differentiation of cultured neurons

**Figure 1:**
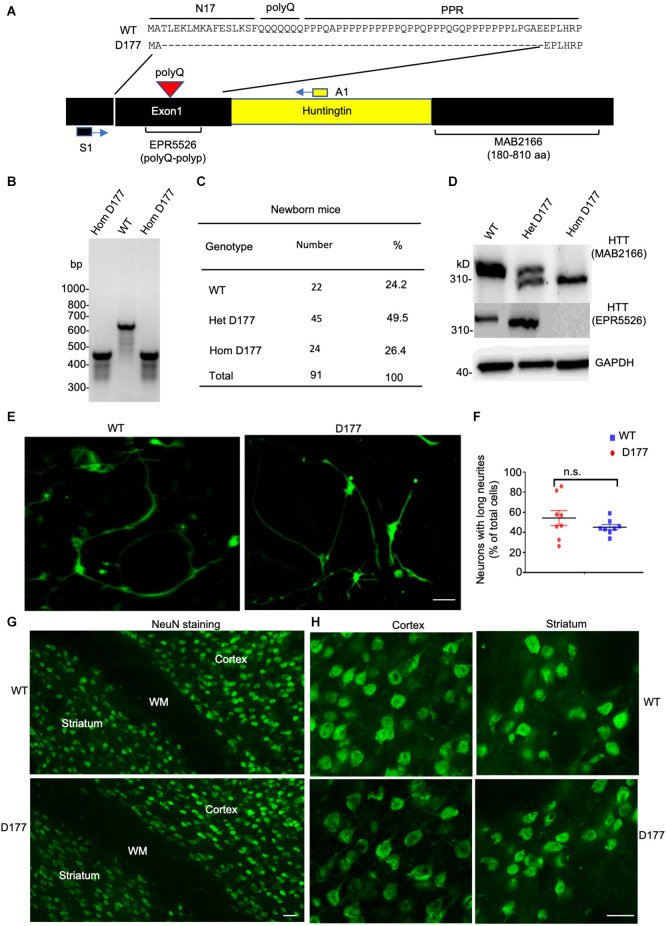
Deletion of *Huntingtin (Htt)* exon 1 in D177 mouse. **(A)** The amino acid sequence of *Htt* exon 1 in D177 and wild-type mice. Note that exon 1 contains a polyQ repeat. Primers [S1 in exon (black) and A1 in intron (yellow)] for PCR and epitopes of antibodies used for Western blotting are indicated. **(B)** The genotyping result of D177 homozygous and wild-type mice using PCR primers indicated in **(A)**. **(C)** The survival rate of newborn mice of WT, heterozygous or homozygous D177 genotype. **(D)** Western blotting showing that the brain cortex of heterozygous D177 mouse expressed the wild-type Htt and a mutated Htt lacking exon 1 whereas homozygous D177 mouse brain only expressed mutated Htt. Note that antibody MAB2166 recognizes both wild type and mutated Htt whereas EPR5526 only reacts with the wild type Htt. **(E)** Cultured primary neurons of wild-type and D177 homozygous mice. The striatal neurons, which were obtained from postnatal day 1 mouse and cultured for 14 days, were labeled with an antibody to TUJ1 to show neuronal processes. Scale bar: 20 μm. **(F)** Percentage of neuronal cells with long neurites (> two times of cell body), *t* = 1.126, df = 7, *n* = 8, *p* = 0.2975. ns = not significant, Student’s *t*-test was used for statistical analysis. Data are presented as mean ± SEM. Image data were collected by ImageJ and were analyzed by GraphPad Prism 7.04. PolyQ, polyglutamine domain, N17, N-terminal 17 amino acids domain; PRR, proline rich region. **(G,H)** NeuN immunohistochemical staining of the brains in 6-month-old WT and homozygous D177 mice at low **(G)** and high **(H)** magnification. WM, white matter in the corpus callosum. Scale bars: 20 μm.

*Htt* exon 1 deletion (D177) mice were generated *via* CRISPR/Cas9 system to target the exon 1 of mouse *Htt* gene, resulting in a deletion of 177 bp, with no reading frame shifting (Figure 1A, Yang et al., 2020). The deletion region includes N17, poly Q and polyproline rich region (PRR). DNA gel analysis of PCR-amplified exon 1 of *Htt* verified that the D177 homozygous mouse has a deletion of 177 bp in the *Htt* DNA (Figure 1B). Homozygous D177 offspring showed the expected mendelian ratio (25%) from mating heterozygous D177 mice, and we did not see sex distortion phenotype (Figure 1C). These results indicate that deletion of *Htt* exon 1 did not affect the survival of newborn mice. We then used two antibodies to examine the expression of full-length *Htt* *via* Western blotting analysis. MAB2166, which reacts with Htt (180–810 aa), could equally detect normal full-length Htt and the smaller D177 Htt that has deleted 59 amino acids, whereas monoclonal rabbit EPR5526 that recognizes the N-terminal Gln- and Pro-rich domain (Aviolat et al., 2019) was unable to react with D177 Htt because of exon 1 deletion (Figure 1D).

We then used mice with homozygous deletion of 177 bp of exon 1 (D177) for further examination. To investigate whether deletion of *Htt* exon 1 has any impact on neuronal development, we cultured striatal neurons from postnatal day 1 of homozygous D177 mice. Using the neuron-specific marker TUJ1 to identify cell body and processes, we found that D177 neurons were differentiated in the same manner as WT neurons and their morphology is indistinguishable from that of WT neurons (Figures 1E,F). To further investigate whether exon 1 deletion has any impact on neuronal cells in the adult brain, we performed NeuN immunohistochemical staining of the brains in 6-month old WT and D177 mice. The density and morphology of neuronal cells in the cortex and striatum appear to be similar between WT and D177 mice (Figures 1G,H).

### Subcellular distribution of HTT in WT and D177 mice

**Figure 2:**
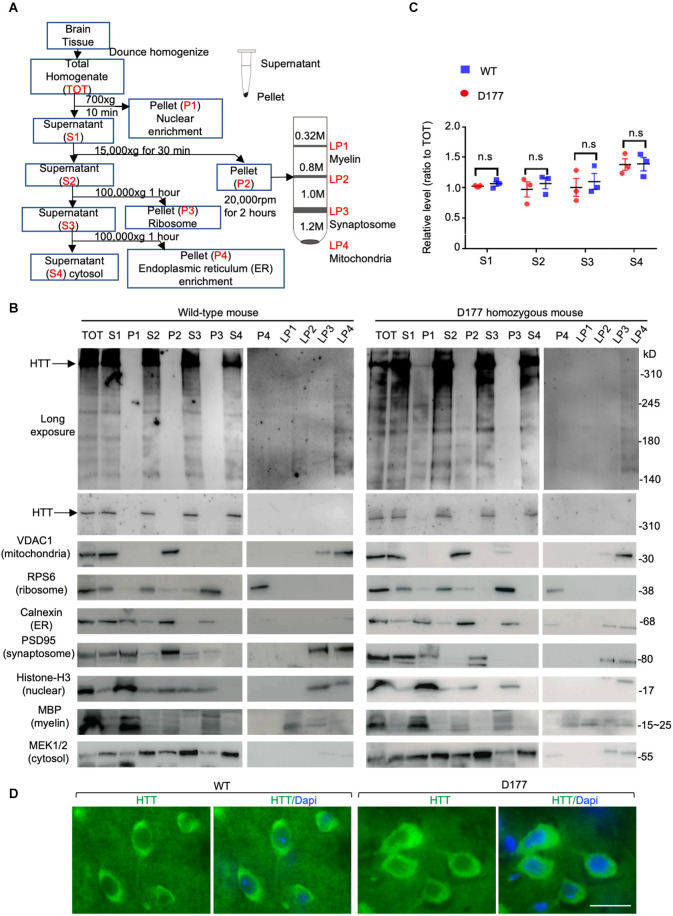
Subcellular distribution of Htt in D177 homozygous and wild-type mice. **(A)** A flowchart method to isolate different subcellular fractions. **(B)** Western blotting analysis of different fractions in the D177 homozygous and wild-type mouse brain, and markers of mitochondria (VDAC1), ribosome (RPS6), ER (Calnexin), synaptosome (PSD95), nuclei (Histone-H3), myelin (MBP), and cytosol (MEK1/2) were examined with their antibodies to confirm the fractionation efficiency. The blots were probed with MAB2166 to detect full-length mouse Htt. Representative Western blots from at least three experiments were presented. Western blots for HTT with short or long exposure time are presented. Other marker proteins are: MEK1/2, mitogen-activated protein kinase 2; Discs Large MAGUK Scaffold Protein 4. **(C)** Relative HTT expression in supernatant fractions of homozygous D177 and wild-type mice (S1, S2, S3 and S4, ratio to TOT), and the expression of HTT was calculated using ImageJ and the values are ratios to “TOT”, *t* = 2.692, df = 3, *n* = 4. ns = not significant. Data were analyzed by GraphPad Prism 7.04. Paired Student’s *t*-test was used for statistical analysis. Data are presented as mean ± SEM. **(D)** HTT immunostaining of the brain cortex in 6-month-old WT and homozygous D177 mice. Anti-HTT (D7F7) was used for staining. Scale bar: 20 μm.

Previous studies have shown that N-terminal mutant HTT with expanded polyQ repeats behaves as a reversible membrane anchor and that polyQ with PPR functions as an interacting region to bind other interacting proteins (Xia et al., 2003; Michalek et al., 2013; Tao et al., 2019; Riguet et al., 2021). How normal Htt is distributed in different subcellular compartments remains to be investigated. Further, it would be important to investigate whether loss of exon 1 can influence its subcellular distribution. We used sucrose density gradient centrifugation to separate different subcellular fractions that are enriched in mitochondrial, ribosomal, ER (endoplasmic reticulum), synaptosomal, nuclear, cytosolic, and myelin proteins (Figure 2A). These fractions were analyzed *via* Western blotting with antibodies to specific organellar marker proteins. Normal full-length Htt and D177 Htt were detected with the anti-HTT antibody (MAB2166). Mouse full-length Htt was cytosolic and mainly distributed in the soluble fractions (S1–S4; Figure 2B). Similar to the normal Htt in wild type (WT) mice, D177 Htt was also mainly distributed in the soluble fractions without significant difference from WT Htt, which was more evident in the blots with a longer exposure time (Figure 2B). Quantification of the relative levels (ratio to total) of Htt in each soluble fraction showed no significant differences between D177 homozygous and wild-type mice (Figure 2C). To validate that HTT is a cytosolic protein, we performed HTT immunohistochemical studies and found that HTT was indeed diffusely distributed in the cytoplasm even in the D177 mouse brain cortex (Figure 2D).

### Expression of autophagy related proteins in D177 mice

**Figure 3:**
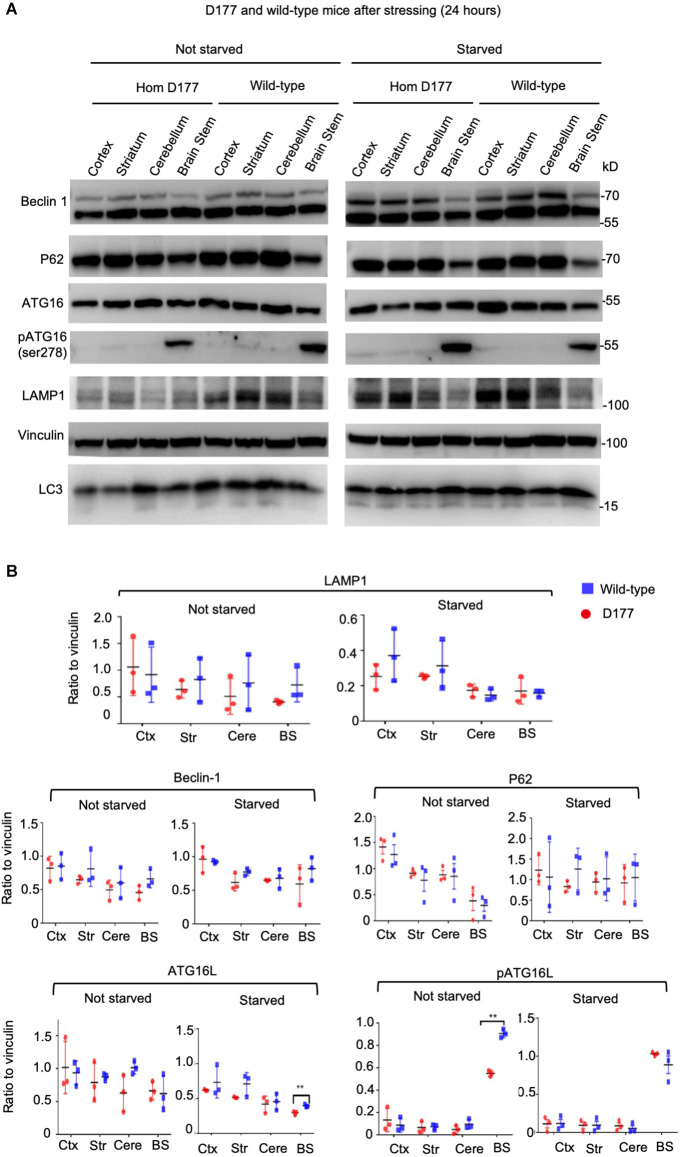
The expression of autophagy related proteins in D177 homozygous and wild-type mice that were not stressed or stressed by food restriction for 24 h. **(A)** Western blotting of autophagy related proteins, Beclin-1, P62, ATG16L, phospho-ATG16L, and LAMP1 in D177 homozygous and wild-type mice with and without stressed by food restriction for 24 h. **(B)** Relative autophagy related protein expression in D177 homozygous and wild-type mice with and without fasting. The values are the ratios of detected protein to the loading control vinculin and were obtained using ImageJ, *n* = 3 mice, df = 4, **P < 0.01. Data were analyzed by GraphPad Prism 7.04 and are presented as men ± SEM. Multiple Student’s *t*-test was used for statistical analysis. p62/SQSTM1, sequestosome 1; ATG16L1, Autophagy Related 16 Like 1; LAMP1, lysosomal associated membrane protein 1; GAPDH, glyceraldehyde-3-phosphate dehydrogenase; Ctx, cortex; Str, striatum; Cere, cerebellum; BS, brain stem.

Full-length Htt has been reported to participate in autophagic function, and mutant Htt can affect autophagy (Martin et al., 2015; Rui et al., 2015; Croce and Yamamoto, 2019). Our previous studies have shown that loss of Htt in adult mouse brains did not alter the expression of P62, an autophagy protein (Wang et al., 2016). However, this finding cannot rule out the role of Htt in regulating autophagy under stress. It is known that starvation can greatly stimulate autophagy in mice (Mizushima et al., 2004). We therefore examined five autophagy related proteins in D177 and wild-type mice under fasting stress condition. WT and D177 mice were deprived of food for 24 h, and their different brain regions (cortex, striatum, cerebellum, and brain stem) were isolated for Western blotting analysis. The Western blotting results showed that most of autophagy-related proteins examined (Beclin 1, P62, ATG16, and pATG16-S278) did not show significant changes between WT and D177 mice with or without starvation (Figures 3A,B). While LAMP1, a lysosomal-associated membrane protein showed a slight reduction in the brain tissues of D177 as compared with WT mice, quantitation of its relative level by measuring its ratio to the loading control vinculin on the same Western blots did not reveal significant difference. pATG16-S278 in the brainstem in WT mice was significantly higher than that of D177 mice without stress, but this difference was vanished under starvation stress. Thus, deletion of exon 1 of *Htt* is unlikely to significantly alter the expression of autophagy-related proteins.

### Gene transcription

HTT has been found to regulate gene expression, and mutant HTT can accumulate in the nucleus to cause significant gene transcription dysregulation in HD mice (Cornett et al., 2005; Truant et al., 2007; Benn et al., 2008; Malla et al., 2021; Gu et al., 2022). To examine whether deletion of Htt exon 1 can alter gene expression, we performed RNA-seq analysis of the cortex, striatum, and cerebellum from three homozygous D177 and four WT mice at 6 months of age, as these three important brain regions show different vulnerability in HD (Ross and Tabrizi, 2011; Bates et al., 2015).

**Figure 4:**
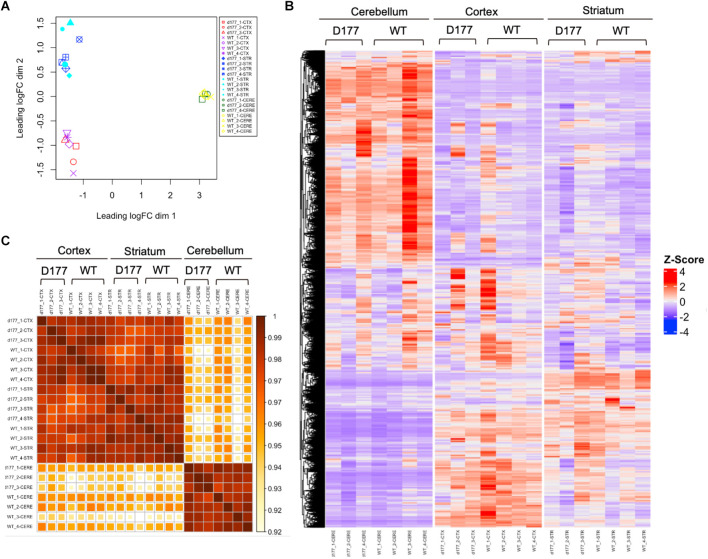
RNA-seq analysis of gene expression in D177 and wild type mice. **(A)** The MDS plot shows that the same brain region samples of D177 and WT mice present low dispersion. **(B)** The Heat map of top 1,000 DEGs (adjust. *P*-value < 0.05, Benjamini–Hochberg, two-sided) upon cortex, striatum, and cerebellum samples of D177 and WT mice. Red indicates increased expression; blue indicates reduced expression. The intensity of the color reflects the degree of gene regulation. **(C)** The corresponding correlation coefficients between each sample are plotted through heatmap. The scale bar indicates correlation coefficients from 0.9 to 1.0.

**Figure 5:**
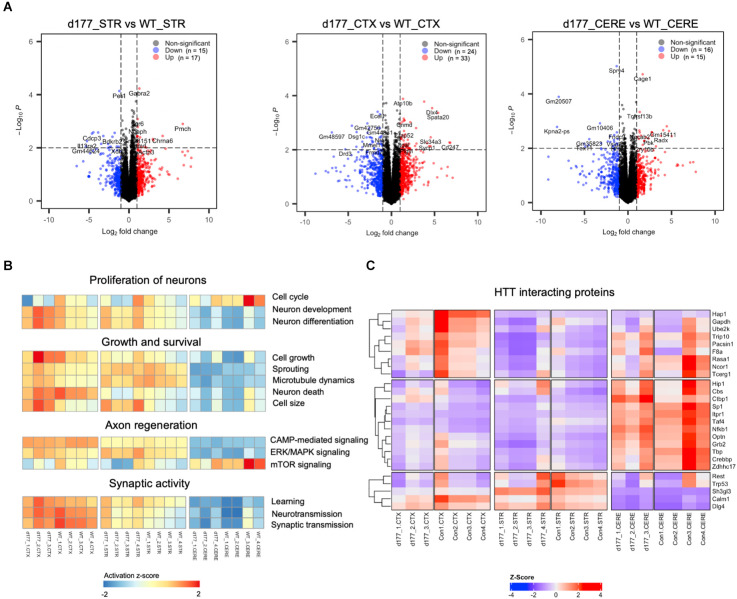
Comparison of gene expression in D177 and wild type mouse brain. **(A)** The volcano plot of DEGs (adjust. *P*-value < 0.05, |fold change| >1.0) in the striatum, cortex, and cerebellum between D177 and WT mice. Red dots indicate up-regulated genes, and blue dots indicate down-regulated genes. **(B)** Gene set variation analysis (GSVA) results suggests that there are few differences between D177 and WT mice in four neuron associated pathway sets. Red indicates pathway activation; blue indicates pathway inhibition. **(C)** The heatmap of HTT directly binding protein expression matrix, the majority of genes show similar mRNA expression levels in the cortex, striatum, and cerebellum between D177 and WT mice.

RNA-seq results showed that the same brain region samples of D177 and WT mice presented short distances from each other and could be clustered in one group in the MDS plot except for one D177 cortex sample (D177_4-CTX) and one D177 cerebellum sample (D177_3-CERE; Figure 4A). However, the heatmap of top 1,000 DEGs (adjust. *P*-value < 0.05, Benjamini–Hochberg, two-sided) in the cortex, striatum, and cerebellum samples of D177 and WT mice did not reveal any obvious differences between D177 and WT (Figure 4B). The corresponding correlation coefficients between each sample were plotted through heatmap, and the brain regional samples in D177 mice samples showed high correlation with the same brain regional samples in WT mice (Figure 4C). The volcano maps also showed no significant differences among the three brain regions, and the differential genes were 17 up-regulated and 16 down-regulated in the striatum, 33 up-regulated and 24 down-regulated in the cortex, and 15 up-regulated and 16 down-regulated in the cerebellum (*p* < 0.01, |Fold change*|* > 1; Figure 5A). To explore the potential neuron-related pathway changes between D177 and WT mice, we performed gene set variation analysis (GSVA) and scored pathway activation degrees in each sample. All four pathway modules including “proliferation,” “growth and survival,” “axonal regeneration,” and “synaptic activity” did not show significant differences in the samples from the same brain region between D177 and WT (Figure 5B). Because HTT can interact with various proteins (Xia et al., 2003; Michalek et al., 2013; Tao et al., 2019; Riguet et al., 2021), we also analyzed the expression of HTT-interacting proteins including Hip1, Sp1, and Hap1. Lacking exon1 in HTT prevented us from performing immunopreciptiation of HTT in D177 mice using HTT antibodies whose epitopes are in the exon 1 region or whose reaction may be modulated by exon 1. Although we could not examine HTT interacting proteins using HTT immunoprecipitation with available anti-HTT antibodies, it would be interesting to see whether depletion of exon 1 in HTT influences the expression of HTT-interacting proteins that can regulate HTT’s function. The heatmap of HTT-interacting protein genes showed that the majority of HTT-interacting protein genes displayed similar mRNA expression levels in the cortex, striatum, and cerebellum between D177 and WT mice (Figure 5C). Taken together, RNA-seq analysis did not reveal significant or substantial alterations in gene expression profiling in D177 mice in which exon 1 *Htt* had been removed.

## Discussion

N-terminal HTT encoded by exon 1 has been found to be important for membrane anchoring, protein interactions, and subcellular distribution (Michalek et al., 2013; Tao et al., 2019; Riguet et al., 2021). In the exon 1 domain of HTT, the first 1–17 amino acids can modulate mutant HTT oligomerization, toxicity, and subcellular localization (Cornett et al., 2005; Gu et al., 2009; Arndt et al., 2019; Chiki et al., 2021; Vieweg et al., 2021). However, our findings showed that removing exon 1 of mouse *Htt* did not significantly alter its subcellular distribution. In support of this, autophagy protein expression and gene expression profiling were not noticeably altered in mice that lack exon 1 of *Htt*. Thus, although exon 1 can modulate the toxicity of mutant HTT, removing this exon 1 does not influence the essential function of wild type Htt in mice. In accordance with this, mice lacking exon 1 *Htt* can live normally without significant changes in growth and behaviors (Neveklovska et al., 2012; Yang et al., 2020; Braatz et al., 2021).

During neuronal development, Htt participates in early brain development and is crucial for establishing neuronal identity, especially in the cortex and striatum (Reiner et al., 2003; Cattaneo et al., 2005; Dragatsis et al., 2018). In the early development process of brain, Htt is localized at spindle pore and regulates spindle orientation and cell fate of cortical progenitors in mitosis (Godin et al., 2010). In addition, Htt also functions in cell migration and anti-apoptosis during neuronal development (Tong et al., 2011). Recent studies of HTT’s function in early brain development were focused on the differences between mutant and normal HTT, uncovering that mutant HTT can affect progenitor cell polarity and differentiation to cause abnormal ciliogenesis and changes in mitotic process (Godin et al., 2010; Elias et al., 2014; Molero et al., 2016; Barnat et al., 2020). Since lacking *Htt* exon 1 does not affect the viability of newborn mice, it is possible the above described functions are not critically dependent on the presence of N-terminal exon 1 domain. Our findings suggest that other domains in HTT should be investigated to explore whether they are associated with the role of HTT during early development.

Our findings provide important implications for HD treatment. First, they support the gain-of-function of exon 1 of HTT in HD pathogenesis. In HD patients, toxic N-terminal HTT fragments are produced by proteolytic cleavage, RNA splicing or RNA translation (Wellington et al., 2000; Sathasivam et al., 2013; Fienko et al., 2022). Although exon 1 HTT may not be essential to cellular function under physiological conditions, it can execute toxic gain-of-function when it carries an expanded polyQ domain, which has been demonstrated by a great number of *in vitro* and *in vivo* studies (Ross and Tabrizi, 2011; Saudou and Humbert, 2016). In support of this idea, we found that normal mouse Htt, unlike polyQ-expanded HTT that can associate with different types of organelles, remains soluble in different cytoplasmic fraction. The implication of our findings is that removing the entire exon 1 in *HTT* gene could be an effective therapeutic strategy. This is because replacing the expanded repeats in exon 1 by a normal repeat in the HTT gene is restricted by the low efficiency of current gene editing tools. It is also challenging to selectively eliminate the expanded allele without disrupting the normal allele of the HD gene. Our findings suggest that removing HTT exon 1 or disrupting exon 1, which can be more readily achieved than knock-in-mediated replacement, does not significantly affect the essential function of HTT in mice. These findings would open a new opportunity to treat HD by simply removing N-terminal exon 1 of mutant HTT.

## Data Availability Statement

The original contributions presented in the study are publicly available. This data can be found here: https://www.ncbi.nlm.nih.gov/sra/?term=PRJNA886100.

## Ethics Statement

The animal study was reviewed and approved by the Animal Care and Use Committee at Jinan University.

## Author Contributions

X-JL and SL designed the research. XZ, YS, and ZW performed the research. XZ, X-JL, SL, and LC analyzed the data. XZ and X-JL wrote the article. All authors contributed to the article and approved the submitted version.

## Funding

This work was supported by The National Natural Science Foundation of China (81830032, 31872779, 82071421), Guangzhou Key Research Program on Brain Science (202007030008), Department of Science and Technology of Guangdong Province (2021ZT09Y007, 2020B121201006), and the high-performance public computing service platform at Jinan University.
